# Exploiting Human CD34^+^ Stem Cell–conditioned Medium for Tissue Repair

**DOI:** 10.1038/mt.2013.194

**Published:** 2013-10-15

**Authors:** Paul J Mintz, Kai-Wen Huang, Vikash Reebye, Georgios Nteliopoulos, Hong-Shiee Lai, Pal Sætrom, Noriyuki Kasahara, Steen Jensen, Madhava Pai, Myrtle YA Gordon, Stephen B Marley, Rosemary Behan, Duncan R Spalding, Abdelali Haoudi, Mohamed M Emara, Joanna Nicholls, John J Rossi, Nagy A Habib

**Affiliations:** 1 Department of Surgery and Cancer, Faculty of Medicine, Imperial College London, London, UK; 2 Qatar Biomedical Research Institute, Education City, Doha, Qatar; 3 Department of Surgery & Hepatitis Research Center, National Taiwan University Hospital, Taipei City, Taiwan; 4 Graduate Institute of Clinical Medicine, National Taiwan University, Taipei City, Taiwan; 5 Department of Haematology, Faculty of Medicine, Imperial College London, London, UK; 6 Department of Cancer Research and Molecular Medicine, Norwegian University of Science and Technology, Trondheim, Norway; 7 Department of Computer and Information Science, Norwegian University of Science and Technology, Trondheim, Norway; 8 Department of Medicine, UCLA School of Medicine, Los Angeles, California, USA; 9 Qatar Biomedical Research Institute, Education City, Doha, Qatar; 10 Department of Virology, School of Veterinary Medicine, Cairo University, Cairo, Egypt; 11 Division of Molecular Biology, Beckman Research Institute of City of Hope, Duarte, California, USA

## Abstract

Despite the progress in our understanding of genes essential for stem cell regulation and development, little is known about the factors secreted by stem cells and their effect on tissue regeneration. In particular, the factors secreted by human CD34^+^ cells remain to be elucidated. We have approached this challenge by performing a cytokine/growth factor microarray analysis of secreted soluble factors in medium conditioned by adherent human CD34^+^ cells. Thirty-two abundantly secreted factors have been identified, all of which are associated with cell proliferation, survival, tissue repair, and wound healing. The cultured CD34^+^ cells expressed known stem cell genes such as Nanog, Oct4, Sox2, c-kit, and HoxB4. The conditioned medium containing the secreted factors prevented cell death in liver cells exposed to liver toxin *in vitro* via inhibition of the caspase-3 signaling pathway. More importantly, *in vivo* studies using animal models of liver damage demonstrated that injection of the conditioned medium could repair damaged liver tissue (significant reduction in the necroinflammatory activity), as well as enable the animals to survive. Thus, we demonstrate that medium conditioned by human CD34^+^ cells has the potential for therapeutic repair of damaged tissue *in vivo*.

## Introduction

Somatic stem cells have the capacity for self-renewal and multilineage differentiation.^1,2,3^ They reside in various tissue types including the bone marrow, brain, skin, muscle, pancreas, and liver.^4^ Studies have shown that soluble factors secreted within the tissue microenvironment including cytokines, and growth factors are essential in stem cell regulation and development.^5,6,7,8,9,10^ They are particularly important in maintaining the homeostasis of their microenvironment, in addition to maintaining a crosstalk with surrounding stromal cells (fibroblasts, endothelial cells, and macrophages). Not surprisingly, emerging evidence suggests that cytokines can also influence stem cell fate *in vitro*, as well as *in vivo*.^11^ Although genes associated with stem cell regulation and development have been studied,^12^ the impact of cytokines and growth factors associated with these regulatory events is less well characterized.

Human CD34^+^ cells are the most widely studied adult stem cells.^12,13^ They reside in the bone marrow, as well as in other tissues and play an essential role in sustaining the formation of the blood and immune systems.^,^^12,14,15,16^ More recently, their proposed ability to differentiate into multiple tissue lineages has been exploited for the treatment of various debilitating diseases.^17,18,19,20^ Studies have shown that CD34^+^ cells are influenced by cytokines and growth factors, in addition to adhesion proteins such as integrins.^21^ However, only a few studies currently show the benefit *in vivo* of factors secreted by human CD34^+^ cells on damaged tissue.

We have previously characterized a population of adherent CD34^+^ cells and based on morphology, flow cytometry, and gene expression analysis they expressed genes corresponding to lineages of tissue differentiation (liver, pancreas, heart, and nerve), as well as hematopoiesis.^18^ Importantly, they also expressed stem cell genes including Oct4, Nanog, and Rex-1. In order to identify the factors secreted by these cells, we have approached this issue by performing a cytokine/growth factor microarray analysis of factors secreted by adherent CD34^+^ cells cultured in a defined serum-free medium. Several abundantly secreted factors were identified from the conditioned medium. Moreover, the conditioned medium prevented cell death in cells treated with a liver toxin *in vitro*, as well as demonstrating tissue repair properties in two independent animal models of liver damage. Our studies suggest that medium conditioned by human CD34^+^ cells may be exploited for therapeutic applications.

## Results

### Adherent human CD34^+^ cells cultured in a defined serum-free medium

**Figure 1:**
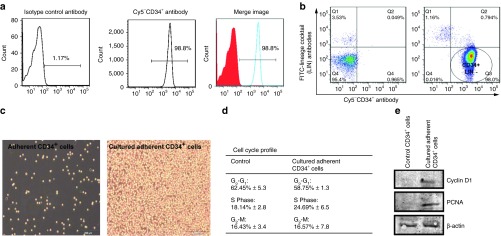
**Characterization of human CD34^+^ cells cultured in a defined serum-free medium.** (**a**,**b**) Histograms showing CD34^+^ cell purity. The *x* axis shows CD34-Cy5 and the *y* axis shows lineage cocktail antibodies (LIN) conjugated to fluorescein isothiocyanate (FITC). The LIN cocktail of antibodies include: CD3, CD14, CD19, CD20, CD56, CD11b, CD10, CD235a, CD7, CD8a, and CD2. Gating was determined by setting a 1% above the isotype control antibody to be considered positive staining. (**c**) Phase-contrast bright field images of 7-day–cultured adherent CD34^+^ cells in the defined serum-free medium. Representative images from four independent experiments. Bar scale, 100 µm. (**d**) Cell cycle profile of 7 day culturing of adherent CD34^+^ cells and K562 cells was used as a positive control. (**e**) Immunoblots of cyclin D1 and PCNA proliferation markers.

To better understand which cytokines and growth factors are secreted by human CD34^+^ cells *in vitro*, we have used a defined serum-free medium in which to grow these cells.^22^ A pure population of isolated human CD34^+^ cells were used for the studies (**Figure 1a**,**b**). Adherent human CD34^+^ cells were grown in a defined serum-free medium containing interleukin (IL)-3, IL-6, and stem cell factor for 7 days as previously described.^22^ The CD34^+^ cells initially form small clusters that gradually merge into a homogenous cell population (**Figure 1c**). We found that the cells were proliferating as determined by the cell cycle profile (**Figure 1d**) and by protein expression of proliferation markers, Cyclin D1 and PCNA (**Figure 1e**). These data suggest that the combination of the three cytokines in the serum-free medium is able to sustain CD34^+^ cell growth and proliferation.

### Expression of cell surface and stem cell markers

**Figure 2:**
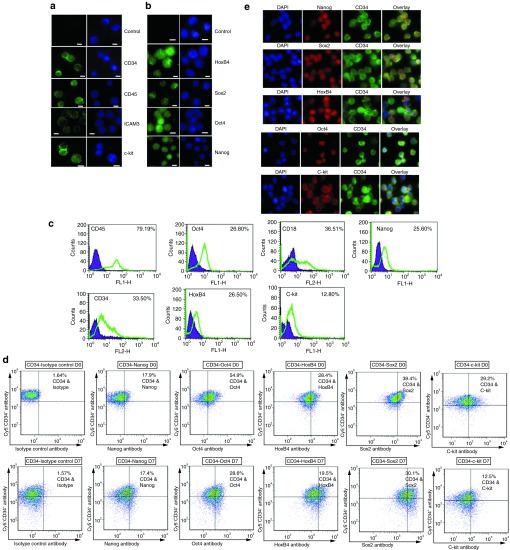
**Analysis of cell surface and stem cell markers.** (**a**) Immunofluorescence staining for cell surface markers for CD45, CD34, ICAM3, and c-kit. Bar scale, 5 µm. The left panel is the antibody stain (green color) and the right panel is the DAPI stain for the nucleus (blue color). Representative images from three independent experiments. (**b**) Immunofluorescence staining for cell markers for HoxB4, Sox2, Oct4, and Nanog. Bar scale, 5 µm. The left panel is the antibody stain (green color) and the right panel is the DAPI stain for the nucleus (blue color). Representative images from three independent experiments. (**c**) Cytometric analysis of the cultured cells for the expression of CD45, CD34, c-kit, CD18, Oct4, Nanog, and HoxB4. Channel gates: FL1-H detects fluorescein isothiocyanate–conjugated antibody and FL2-H detects phycoerythrin-conjugated antibody. Gating was determined by setting a 1% above the isotype control antibody to be considered positive staining. Representative images from two independent experiments. (**d**) Double staining of CD34 and stem cell markers. CD34^+^ cells (D0) and 7 days (D7) cultured adherent CD34^+^ cells were stained with the indicated antibodies. Stem cell markers are plotted on the *x* axis; CD34 staining is plotted on the *y* axis. The percentage of double-positive cells is shown in the upper right quadrant. (**e**) Colocalization of CD34 and stem cell markers on the cultured adherent CD34^+^ cells. DAPI, 4′6′-diamidino-2-phenylindole.

We next used flow cytometry and immunostaining approaches to determine whether the cells grown in the defined medium still expressed known cell lineage and stem cell markers. Several important cell surface markers including CD34, c-kit, CD45, and ICAM3 were expressed (**Figure 2a**). In addition to the cell lineage markers, specific stem cell markers including Oct4, HoxB4, Nanog, and Sox2 were expressed positively, in addition to being coexpressed with CD34 (**Figure 2b**–**e**). Stem cell genes such as Oct4, Sox2 and Nanog are essential in stem cell regulation and self-renewal.^2^ Collectively, these results suggest that the CD34^+^ cells cultured in the serum-free defined medium are able to maintain the expression of several important cell lineage and stem cell markers.

### Cytokine expression profile of conditioned medium

**Figure 3:**
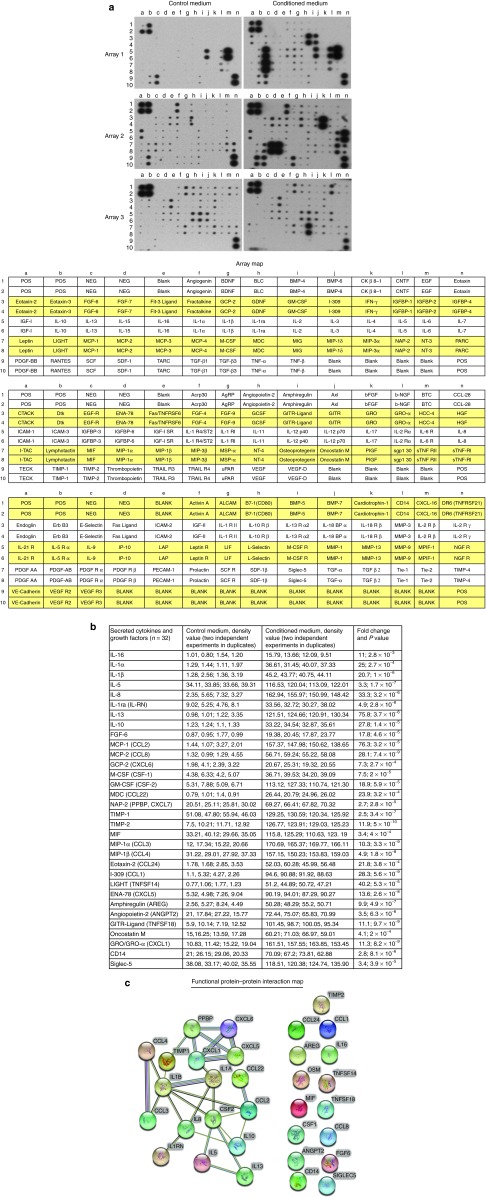
**Cytokine profiling and protein–protein interaction map.** (**a**) A 174 cytokine and growth factor antibody array was probed with control medium (defined medium without cells) or concentrated (2.5-fold) conditioned medium (defined medium plus adherent CD34^+^ cells). The positive controls are located in the upper left-hand corner (four spots) and lower right-hand corner (two spots) for each membrane. The array contains duplicate spots for each cytokine or growth factor. Representative images from two independent experiments. A map of the cytokines and growth factors are listed on the adjacent side of each array. (**b**) Cytokines and growth factors that had 1.5-fold or more relative to the control were scored as positive. A total of 32 factors were scored positive. Densitometry was used to quantify the relative intensity of the spots to the control. (**c**) A protein–protein interaction network map for the 32 secreted factors was generated using the STRING program. The STRING program covers over 2.5 million proteins. The confidence score was set at the highest level, >90%.

In order to identify the secreted factors, the conditioned and control mediums were analyzed using a 174 cytokine antibody microarray chip for secreted soluble factors. Of the numerous factors represented on the array (**Figure 3a**), 32 were significantly abundant in the conditioned medium compared with the control medium (**Figure 3b**). Some of the identified cytokines and growth factors such as FGF-6, IL-8, IL-10, M-CSF, GM-CSF, and angiopoietin-2 are important in stem cell regulation, as well as in cell proliferation and tissue repair.^6,9,11^ To gain further insight into how the secreted factors are interconnected with each other, we performed a bioinformatic analysis for protein–protein interactions using a database called STRING (Search Tool for the Retrieval of Interacting Genes/Proteins).^23,24^ This database examines multiple genes and proteins simultaneously for physical and functional interactions based on seven different criteria for known individual factors. Using this program, a predicted functional protein–protein interaction map was generated between the different secreted factors (**Figure 3c**). The protein–protein interaction map revealed that 17 out of the 32 secreted factors have some degree of multiple functional interactive partners. A closer analysis of these 17 factors for molecular and cellular functions revealed that several of them are important for cell proliferation, wound healing, and immune response (**Table 1**). These molecules frequently have overlapping activities and can act in an autocrine or paracrine fashion. This is not surprising because a complex network of growth factors and cytokines are essential for cellular differentiation and tissue regeneration.

### Conditioned medium prevents cell death in liver cells

**Figure 4:**
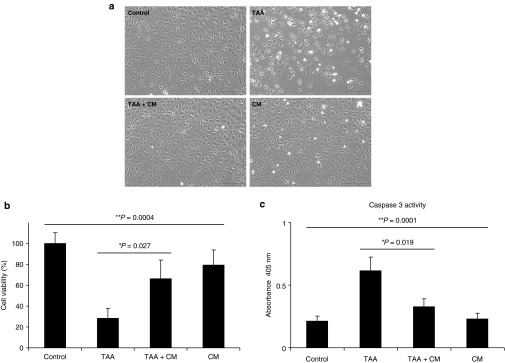
**Effect of the conditioned medium (CM) on damaged liver cells treated with thioacetamide (TAA).** Rat liver cells with TAA displayed a (**a**) structural damage as well as (**b**) reduced cell viability and (**c**) increased caspase-3 activity as determined by ELISA assays. Error bar represent mean ± SD (*n* = 3). *Student's *t*-test. **Analysis of variance test (includes all the different groups). ELISA, enzyme-linked immunosorbent assay.

The information from the protein–protein interaction map lead us to consider the possibility that the conditioned medium may have protective and wound healing properties based on what is already known about some of the secreted factors. As it is complex and technically challenging to test all of the 32 secreted factors individually or in various combinations, we decided to directly test the conditioned medium. We found that the conditioned medium can prevent cell death in liver cells treated with a liver toxin, thioacetamide (TAA) (**Figure 4a**). We found that liver cells treated with TAA displayed severe structural damages, whereas TAA-treated cells that received the conditioned medium maintained normal structural integrity, as well as cell viability (**Figure 4a**,**b**). As it is known that TAA activates the caspase-3 signaling pathway,^25^ we tested for caspase-3 activity under these conditions. We observed a reduced level of caspase-3 activity in liver cells treated with the conditioned medium (**Figure 4c**). These results suggest that the conditioned medium contains secreted factors that may prevent cell death.

### Conditioned medium repairs liver damage *in vivo*

**Figure 5:**
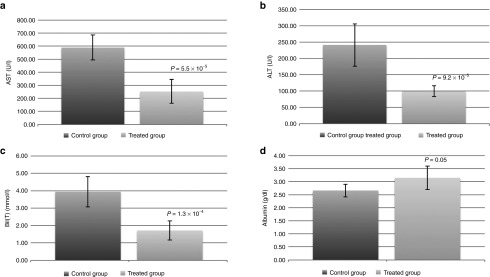
**Effect of the conditioned medium on damaged liver tissue *in vivo*.** (**a**) Alanine transaminase (ALT), (**b**) aspartate aminotransferase (AST), and (**c**) Bil (bilirubin) all showed reduced levels in the treated group. (**d**) Albumin showed slight increase level in the treated group. Statistical significance: AST, *P* = 5.5 × 10^−5^; ALT, *P* = 9.2 × 10^−5^; Bil, *P* = 1.3 × 10^−4^; Albumin, *P* = 0.05. The treated and control groups contained eight rats per group.

**Figure 6:**
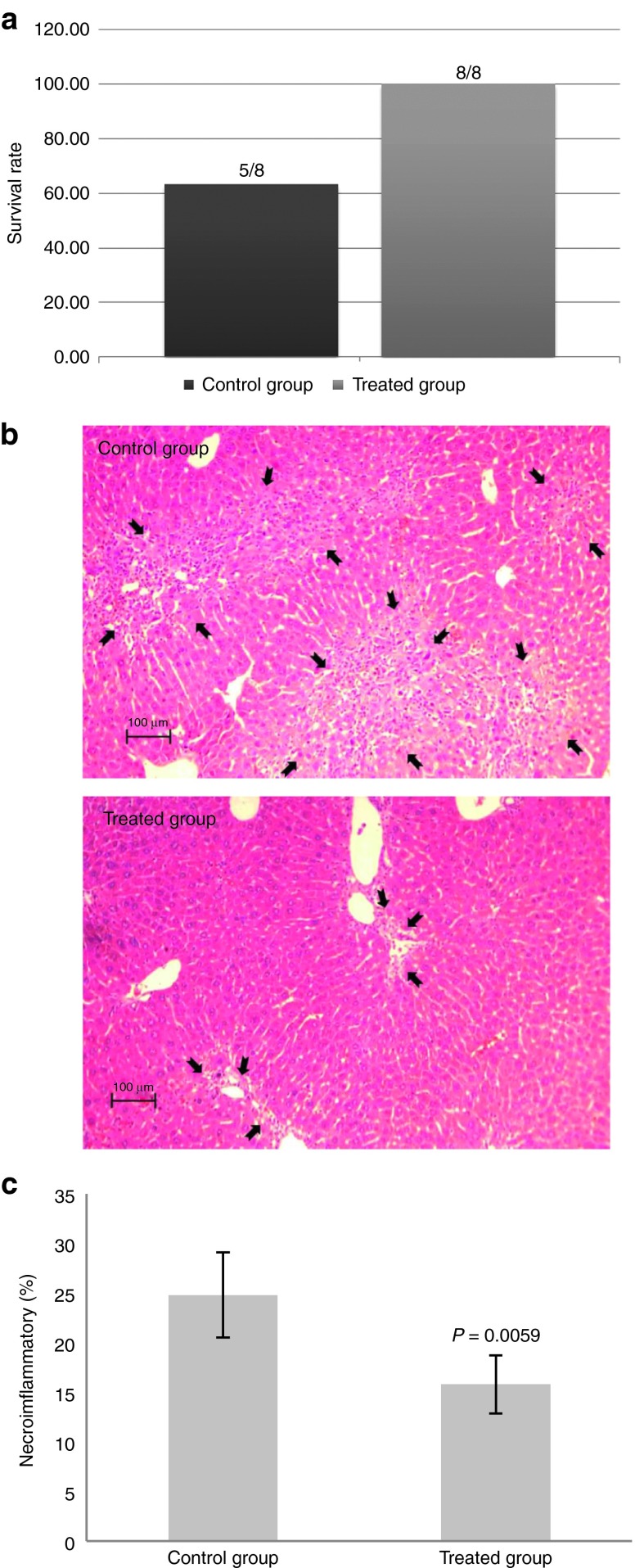
**Necroinflammatory activity of liver tissue treated with the conditioned medium.** (**a**) Survival outcome in the treated group (*n* = 8/8) compared with the control group (*n* = 5/8). (**b**) Hematoxylin and eosin staining of liver tissue from the treated and control group. Necroinflammatory areas are indicated by black arrows. Scale bar: 100 μm. (**c**) Necroinflammatory quantification. Approximately 24.8 ± 4.28% of the liver developed necroinflammatory activity in the control group versus 15.8 ± 2.93% in the treated group. Statistical significance: control versus the treated group, *P* = 0.0059.

Based on the results from the *in vitro* studies (**Figure 4**) and information from **Table 1**, we next investigated the effect of the conditioned medium in a rat liver model. Specifically, we used a clinically relevant cirrhotic rat liver model which demonstrates features similar to human liver cirrhosis.^26^ The induced cirrhotic rats were injected six times via tail vein with the conditioned medium and killed 3 days after the last injection. Important liver functions including aspartate aminotransferase, alanine transaminase, bilirubin, and albumin were measured. We found that animals treated with the conditioned medium performed much better for all the liver functions when compared with the control group (**Figure 5a**–**d**). More importantly, all the animals survived in the treated group (**Figure 6a**), suggesting that the conditioned medium has the capacity to extend the survival outcome of the treated animals. To validate this observation, we specifically determined the amount of necroinflammatory activity in the damaged liver tissue. The measurement of its activity reflects the degree of necrosis and inflammation in the liver. The staining revealed a reduced necroinflammatory area (**Figure 6b**,**c**) in the treated group (15.8 ± 2.93%) compared with the control group (24.8 ± 4.28%). As these results suggest that the conditioned medium may have the capacity to repair damaged liver tissue, we next determined whether the cells would also have a similar affect. Surprisingly, when 7-day–cultured cells were injected into rats that were pretreated with a well-established liver toxin, TAA, we found improved albumin and bilirubin liver functions (**Supplementary Figure S1**).

## Discussion

There is a high expectation that stem cells may deliver new therapeutic strategies for debilitating diseases such as neurodegenerative conditions, diabetes, cardiovascular disorders, and liver disease.^26^ Tremendous effort has been invested in developing new stem cell–based therapeutic products using embryonic and adult stem cells for regenerative medicine. In addition to exploiting stem cells themselves as the deliverable therapeutic agent, the idea of exploiting the factors secreted by stem cells has been less characterized.

The approach we have taken, using a defined serum-free medium to culture human CD34^+^ cells, has enabled the identification of several enriched secreted factors. More importantly, our study is the first to demonstrate that the medium conditioned by adherent CD34^+^ cells are able to reach the damaged liver and repair tissue in a clinically relevant liver animal model. We found a significant reduction in the degree of necrosis and inflammation (necroinflammatory activity) in the liver treated with the conditioned medium, suggesting that the factors in the conditioned medium are capable of reducing the inflammatory response. We speculate that under these conditions, it is possible that the secreted factors in the conditioned medium may provide the necessary survival and repair factors to recruit various cell types and to assist in the tissue repair process. In accordance with this idea, studies have shown that factors secreted from stem cells may be associated with vascular repair and regeneration.^27,28^ It is interesting to note that many of the identified factors secreted by human CD34^+^ cells are involved in cell survival and wound healing. For example, the protein–protein interaction map revealed four highly interactive networks of chemokines (CXCL1, CXCL5, CXCL6, and PPBP) that belong to CXC ligand (CXCL) chemokine family, which is well known to recruit neutrophils to the wound site and actively aid in the repair process.^29^

The healing properties of this family have been expanded to include an efficient repair and regeneration of mice liver with partial hepatectomy.^29^ There is growing evidence that this hepatic repair process is regulated by CXC chemokine receptor 2 (CXCR2),^30,31^ which commonly binds to members of the CXCL chemokine family,^32,33^ suggesting that the four chemokines may play a major role in repairing injured liver cells but further studies are needed.

In summary, we have shown proof-of-concept that factors secreted by human CD34^+^ cells in the conditioned medium have tissue repair properties. If this approach proves to be successful, the secreted factors may be developed into a clinical product that could be readily processed and stored for therapeutic applications.

## Materials and Methods

***Cell culture.*** Normal rat liver cell line CRL-1439 was obtained from American Type Culture Collection (Rockville, MD) and cultured in RPMI supplemented with 10% fetal bovine serum.

***Isolation and growth of adherent CD34^+^ cells.*** Allogeneic human hematopoietic donor blood samples were obtained with informed patient consent and approved by the Hammersmith Hospital Research Ethics Committee. Samples of granulocyte-colony stimulating factor (G-CSF) mobilized peripheral blood progenitor cells were processed by leukapheresis at the Stem Cell Laboratory at the Hammersmith Hospital. Briefly, human mobilized peripheral blood samples were diluted in a ratio of 1:4 in Hanks' buffered saline solution (Invitrogen, Paisley, UK), the mononuclear cells were separated by centrifugation over a Lymphoprep (Axis-Shield, Dundee, Scotland) density gradient at 1800 rpm for 30 minutes. The mononuclear cell fraction at the interface was aspirated and washed twice with Hanks' buffered saline solution, and finally with magnetic cell sorting buffer (phosphate-buffered saline solution at pH 7.2 supplemented with 0.5% bovine serum albumin and 2 mmol/l EDTA). CD34^+^ cells were isolated using a CD34^+^ isolation kit (Miltenyi Biotec, Surrey, UK) according to the manufacturer's protocol. The purity of CD34^+^ cells were determined by FACS analysis and only samples that were >95% pure were used for the studies. Flow cytometry analysis of isolated CD34^+^ cells were confirmed by the following antibodies: CD34-PerCP-Cy5 (1:300); lineage cocktail antibodies CD3, CD14, CD19, CD20, CD56, CD11b, CD10, CD235a, CD7, CD8a, and CD2 conjugated to fluorescein isothiocyanate. Directly conjugated isotype-matched controls (1:300) (IgG1κ, IgG2a; BD Bioscience, Oxford, UK) were used as negative controls, while Fluorescence Minus One was used alternatively. Stained cells were analyzed using the BD LSRII flow cytometer with 488–530/30, 488–695/40, and 488–780/60 filters to view fluorescein isothiocyanate and PerCP-Cy5.5 respectively. At least 10,000 cells were collected for each test sample to ensure a sufficient number of positive stained cells. FlowJo 7.5 software was used for the presentation of the results. The isolated CD34^+^ cells were added to 24-well or 35-mm tissue-culture–treated dishes (Nunc) (Sigma-Aldrich Company, Dorset, UK) at a density of 2.5–5 × 10^5^ cells in α-minimum essential medium medium to isolate the adherent CD34^+^ cells. After 30 minutes incubation, cells were rinsed and grown in a defined serum-free medium (CellGro; CellGenix GmbH, Freiburg, Germany) containing three cytokines: 250 ηg/ml of stem cell factor, 250 ηg/ml of IL-6, and 250 ηg/ml of IL-3 (Invitrogen or CellGenix GmbH) in 0.5% penicillin/streptomycin antibiotics. Cells were incubated at 37 °C in 5% CO_2_.^22^ Approximately 1 ml of the defined serum medium was added when culturing in 35-mm culture plates and 500 µl per well when 24-well plates were used. Total viable cells were counted using the trypan exclusion assay.

***Cytokine expression profiling.*** The isolated adherent CD34^+^ cells (35-mm dishes, Nunc) (Sigma-Aldrich Company) were cultured in the defined serum-free medium (5 plates per array). The control dishes only contained the defined serum-free medium. The conditioned medium was collected on the 3rd and 7th days. In order to prevent any cells remaining in the conditioned medium, the medium was centrifuged for 1 hour at 13,000 RPM at 0 °C. The conditioned media were pooled and concentrated to 2 ml using a Vivaspin-20 concentrator (Sigma-Aldrich Company). The concentrated medium (2.5-fold) was used for the RayBio human cytokine antibody array C-series 2000 (174 cytokines). Briefly, membranes were blocked (blocking buffer) followed by adding the conditioned medium or control medium. After 2 hours of incubation, membranes were repeatedly washed and then incubated with biotin-conjugated antibodies for 2 hours at room temperature. The membrane was washed followed by horse-radish peroxidase conjugated streptavidin detection for 1 hour followed by several washes. The membranes were processed followed by exposure to hyperfilm (GE Healthcare, Buckinghamshire, UK). The film was processed and scanned, and densitometric value of each spot on the array was measured using the UVP GelDoc system (UVP, Cambridge, UK). Cytokines that had 1.5-fold or more relative to the control were scored as positive. Two independent experiments were performed and analyzed. Each array contained duplicate spots of the cytokines or growth factors. Protein–protein interaction was generated using the STRING (Search Tool for the Retrieval of Interacting Genes/Proteins) database.^23,24^

***Immunofluorescence microscopy, flow cytometry, cell cycle, and western blot.*** To analyze the expression of cell surface and intracellular markers, flow cytometry and immunofluorescence analysis were performed. Approximately 1 × 10^5^ cells were stained and analyzed per sample. The expression of specific stem cell markers was performed on day-7–cultured cells. For cell surface markers, no permeabilization was performed before immunolabeling with the appropriate primary antibodies. For intracellular staining, cells were fixed and permeabilized using Fix & Perm kit (Invitrogen) or 0.2% Triton-X-100 in phosphate-buffered saline followed by labeling with appropriate primary antibodies. Each tube was washed and stained with the appropriate secondary antibody (1:500). Isotype-matched controls were used for each antigen stained. Analysis of the labeled cells was performed with a FACScalibur flow cytometer (BD Bioscience). Ten thousand cells were collected for each test sample to ensure a sufficient number of positive stained cells. For the immunofluorescence staining, after several washes in phosphate-buffered saline, coverslips were mounted on glass slides with Vectashield containing 4′6′-diamidino-2-phenylindole (Vector Laboratories, Peterborough, UK). Slides were visualized on a Leica DM4000 or Zeiss Axiovert 200 inverted fluorescence microscope. Antibodies used were anti-Nanog (1:50 and 1:10) (Santa Cruz Biotechnology, Dallas, TX and Abcam, Cambridge, UK); anti-CD45 (1:200), anti-CD18 (1:100) (BD Bioscience and Abcam); anti-c-kit (1:50 and 1:10) (BD Bioscience and Abcam); anti-CD34 (1:20 and 1:5) (Santa Cruz Biotechnology and Abcam); anti-ICAM3 (1:40) (Abcam); anti-HoxB4 (1:20), anti-Sox2 (1:50 and 1:10) (Abcam and Autogen Bioclear UK, Wiltshire, UK); and anti-Oct-4 (1:100 and 1:15) (Sigma-Aldrich Company and Abcam). Appropriate isotype control antibodies were purchased from BD Biosciences. Gating was determined by setting a 1% above the isotype control antibody to be considered positive staining. For the double staining of CD34 and stem cell markers, the following antibodies were used: CD34-PE-Cy5, c-kit-PE), Nanog-AlexaFluor488, Oct4-AlexaFluor488, HoxB4-488, Sox2-Alexafluor488 and isotype-matched controls (IgG1κ and IgG2a,) were used as negative controls (BD Bioscience and Abcam). Stained cells were analyzed using the BD LSRII flow cytometer with 488–530/30, 488–575/26, and 488–695/40 filters to view Alexa488, PE and PE-Cy5 respectively. For the western blot, all cell extracts were prepared from CD34^+^ cells cultured in the defined growth medium. CD34^+^ cells not cultured in the growth medium were used as control cells. Equal protein concentration of 60 µg were separated by SDS-PAGE. Antibodies used were anti-CyclinD1 (1:100) (New England Biolab, Ipswich); anti-PCNA (1:100) (New England Biolab); and anti-β actin (1:300) (Sigma-Aldrich Company). Propidium iodide staining was used to analyze the cell cycle and K562 cell line was used as a positive control.

**In vitro *TAA studies*.** To test the CD34^+^ cells cultured conditioned medium, rat liver cells were seeded at 2.5 × 10^5^ on 24-well plates. After 24 hour incubation, the cells were washed once with phosphate-buffered saline followed by α-minimum essential medium. The cells were then treated with liver toxin, TAA (0.2 µg/µl) (Sigma-Aldrich Company) or TAA with the conditioned medium (20 µl) in α-minimum essential medium. Standard culturing medium was used a positive control. The treated cells were incubated for 48 hours before performing cell viability and caspase-3 assays. Cell viability was performed with WST-1 kit (Roche, West Sussex, UK) and the caspase-3 activity was determined using Caspase-3 kit (Invitrogen). Cell morphological analysis was determined using an Olympus microscope.

**In vivo *animal studies*.** We used a clinically relevant cirrhotic animal model generated from a modified diethylnitrosamine feeding protocol.^26^ Briefly, male Wistar rats (150–180 g) at 7 weeks of age were obtained from the Animal Center of National Taiwan University. All the experiments were conducted in accordance with the “Guide for the Care and Use of Laboratory Animals” prepared by the Institutional Animal Care and Use Committee of National Taiwan University. The animals were given diethylnitrosamine solution daily for 11 weeks, starting with 100 ppm in the first week. The average body weight of the animals was measured once a week and the concentration of diethylnitrosamine in their drinking water was adjusted in proportion to the body weight each week relative to that of the first week. After 11 weeks, the functional liver insufficiency was determined by the decreased bile flow as previously described.^26^ Liver cirrhosis was determined by irreversible pathological change and collage accumulation in the liver after 9 weeks into the induction. For the *in vivo* study, 100 µl of the conditioned medium of CD34^+^ cells were injected via tail vein per cirrhotic rat in a 2-week period (a total of six injections per rat, eight rats per group). Control animals (*n* = 8) were injected with equal volume of control medium. The rats were killed 3 days after the last injection. Four-micrometer–thick liver sections were deparaffinized and rehydrated. For histological examination, the slides were stained with hematoxylin and eosin. Morphometric analysis was performed measuring the percentage of necrotic areas in 10 fields versus total section area at 200 magnification using the Digital Camera System HC-2500, Adobe Photoshop version 5.0J, and Image-Pro Plus version 3.0.1J (Media Cybernetics, Bethesda, MD).

For the *in vivo* study using the hepatotoxin TAA, rats were exposed to the TAA at 350 mg per Kg body weight, which induces liver damage. Male Wistar rats (*Rattus norvegicus*) were used in this study. Three groups were in the study: group 1 (*n* = 5, control with no TAA), group 2 (*n* = 7, TAA treated), and group 3 (*n* = 7, TAA treated followed by injection of cultured stem cells). The animals were treated with TAA for 2 consecutive days followed by immunosuppressant treatment (cyclosporine) at 0.5 mg/100 g body weight. The following day the rats were injected with 2 × 10^7^-cultured stem cells per rat. After 3 days of treatment, liver tissues and blood samples were taken from all the three groups for analysis. Liver functions were assayed by measuring bilirubin and albumin levels (BioAssay Systems, Hayward, CA). Hematoxylin and eosin staining were performed on rat liver tissues (5 µm sections). All animal experiments were carried out according to the institutional and Home Office guidelines.

***Statistical analysis.*** Statistical analyses were performed using Prism software (version 3.0). Statistical significant differences were determined by Student's *t*-test or one-way analysis of variance test. Data are presented as mean ± SDs.

**SUPPLEMENTARY MATERIAL** **Figure S1.** Effect of the expanded CD34^+^ cells transplanted on damaged rat liver *in vivo*.

## Figures and Tables

**Table 1:**
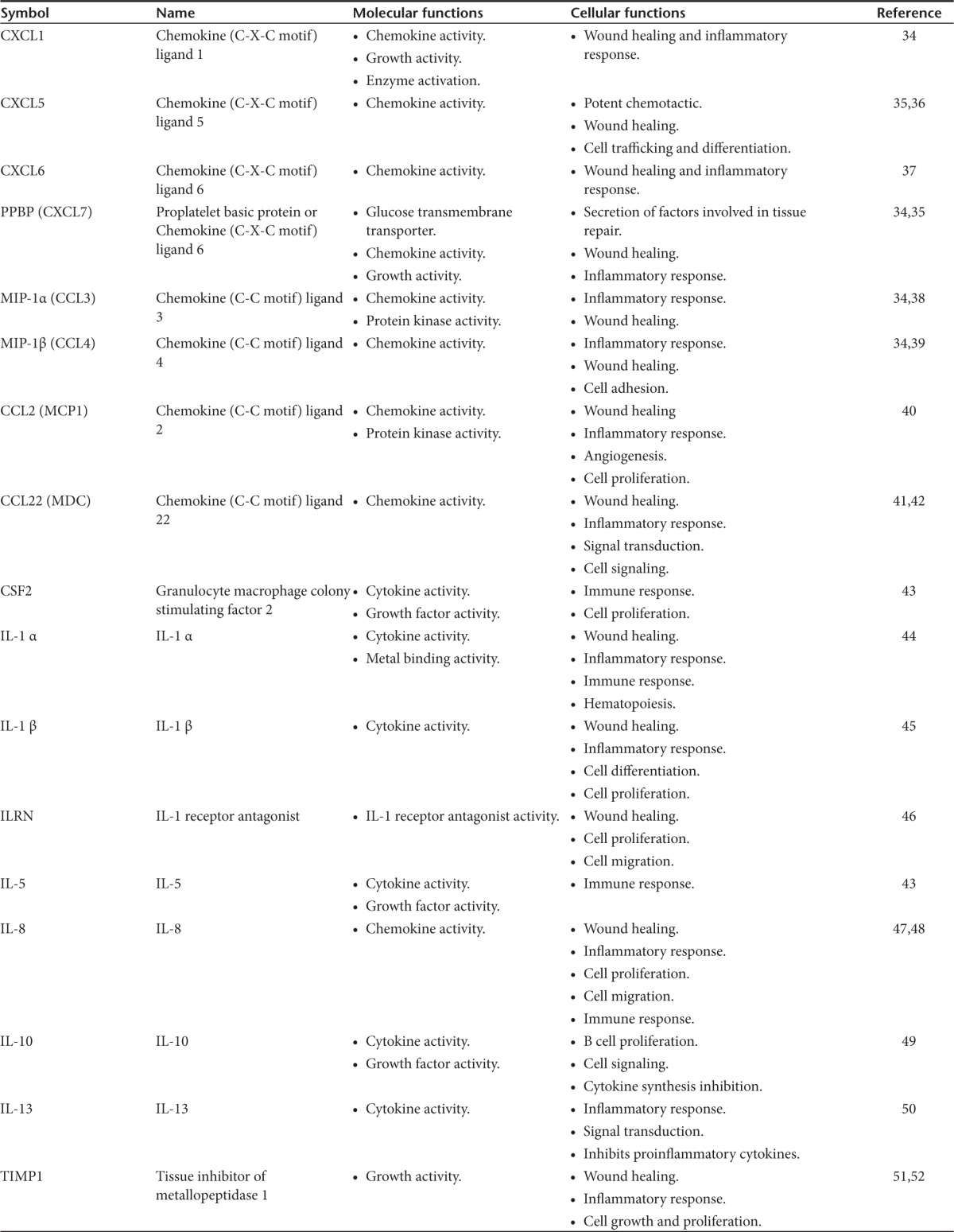
Molecular and cellular functions for the selected secreted factors

## Supplementary Material

Supplementary Figure S1 Effect of the expanded CD34^+^ cells transplanted on damaged rat liver *in vivo*.

## References

[bib1] Weissman IL 2000 Stem cells: units of development, units of regeneration, and units in evolution. Cell 100 157 168 10647940 10.1016/s0092-8674(00)81692-x

[bib2] Jaenisch R Young R 2008 Stem cells, the molecular circuitry of pluripotency and nuclear reprogramming. Cell 132 567 582 18295576 10.1016/j.cell.2008.01.015 PMC4142810

[bib3] Fuchs E Tumbar T Guasch G 2004 Socializing with the neighbors: stem cells and their niche. Cell 116 769 778 15035980 10.1016/s0092-8674(04)00255-7

[bib4] Slack JM 2000 Stem cells in epithelial tissues. Science 287 1431 1433 10688782 10.1126/science.287.5457.1431

[bib5] Janowska-Wieczorek A Majka M Ratajczak J Ratajczak MZ 2001 Autocrine/paracrine mechanisms in human hematopoiesis. Stem Cells 19 99 107 11239164 10.1634/stemcells.19-2-99

[bib6] Majka M Janowska-Wieczorek A Ratajczak J Ehrenman K Pietrzkowski Z Kowalska MA 2001 Numerous growth factors, cytokines, and chemokines are secreted by human CD34(+) cells, myeloblasts, erythroblasts, and megakaryoblasts and regulate normal hematopoiesis in an autocrine/paracrine manner. Blood 97 3075 3085 11342433 10.1182/blood.v97.10.3075

[bib7] Bonin-Debs AL Boche I Gille H Brinkmann U 2004 Development of secreted proteins as biotherapeutic agents. Expert Opin Biol Ther 4 551 558 15102604 10.1517/14712598.4.4.551

[bib8] Zhang CC Lodish HF 2008 Cytokines regulating hematopoietic stem cell function. Curr Opin Hematol 15 307 311 18536567 10.1097/MOH.0b013e3283007db5 PMC2677548

[bib9] Abarbanell AM Coffey AC Fehrenbacher JW Beckman DJ Herrmann JL Weil B 2009 Proinflammatory cytokine effects on mesenchymal stem cell therapy for the ischemic heart. Ann Thorac Surg 88 1036 1043 19699961 10.1016/j.athoracsur.2009.02.093

[bib10] Haylock DN To LB Dowse TL Juttner CA Simmons PJ 1992 Ex vivo expansion and maturation of peripheral blood CD34+ cells into the myeloid lineage. Blood 80 1405 1412 1381625

[bib11] Nervi B Link DC DiPersio JF 2006 Cytokines and hematopoietic stem cell mobilization. J Cell Biochem 99 690 705 16888804 10.1002/jcb.21043

[bib12] Orkin SH Zon LI 2008 Hematopoiesis: an evolving paradigm for stem cell biology. Cell 132 631 644 18295580 10.1016/j.cell.2008.01.025 PMC2628169

[bib13] Kondo M Wagers AJ Manz MG Prohaska SS Scherer DC Beilhack GF 2003 Biology of hematopoietic stem cells and progenitors: implications for clinical application. Annu Rev Immunol 21 759 806 12615892 10.1146/annurev.immunol.21.120601.141007

[bib14] Till JE McCulloch EA 1961 A direct measurement of the radiation sensitivity of normal mouse bone marrow cells. Radiat Res 14 213 222 13776896

[bib15] Bianco P Riminucci M Gronthos S Robey PG 2001 Bone marrow stromal stem cells: nature, biology, and potential applications. Stem Cells 19 180 192 11359943 10.1634/stemcells.19-3-180

[bib16] Kaushansky K 2006 Lineage-specific hematopoietic growth factors. N Engl J Med 354 2034 2045 16687716 10.1056/NEJMra052706

[bib17] Pai M Zacharoulis D Milicevic MN Helmy S Jiao LR Levicar N 2008 Autologous infusion of expanded mobilized adult bone marrow-derived CD34+ cells into patients with alcoholic liver cirrhosis. Am J Gastroenterol 103 1952 1958 18637092 10.1111/j.1572-0241.2008.01993.x

[bib18] Gordon MY Levicar N Pai M Bachellier P Dimarakis I Al-Allaf F 2006 Characterization and clinical application of human CD34+ stem/progenitor cell populations mobilized into the blood by granulocyte colony-stimulating factor. Stem Cells 24 1822 1830 16556705 10.1634/stemcells.2005-0629

[bib19] Levicar N Pai M Habib NA Tait P Jiao LR Marley SB 2008 Long-term clinical results of autologous infusion of mobilized adult bone marrow derived CD34+ cells in patients with chronic liver disease. Cell Prolif 41 suppl. 1 115 125 18181952 10.1111/j.1365-2184.2008.00491.x PMC6496663

[bib20] McNiece I Briddell R 2001 Ex vivo expansion of hematopoietic progenitor cells and mature cells. Exp Hematol 29 3 11 11164100 10.1016/s0301-472x(00)00610-x

[bib21] Suárez-Álvarez B López-Vázquez A López-Larrea C 2012 Mobilization and homing of hematopoietic stem cells. Adv Exp Med Biol 741 152 170 22457109 10.1007/978-1-4614-2098-9_11

[bib22] Mintz PJ Sætrom P Reebye V Lundbæk MB Lao K Rossi JJ 2012 MicroRNA-181a* Targets Nanog in a Subpopulation of CD34(+) Cells Isolated From Peripheral Blood. Mol Ther Nucleic Acids 1 e34 23344176 10.1038/mtna.2012.29 PMC3437805

[bib23] Jensen LJ Kuhn M Stark M Chaffron S Creevey C Muller J 2009 STRING 8–a global view on proteins and their functional interactions in 630 organisms. Nucleic Acids Res 37 Database issue D412 D416 18940858 10.1093/nar/gkn760 PMC2686466

[bib24] Szklarczyk D Franceschini A Kuhn M Simonovic M Roth A Minguez P 2011 The STRING database in 2011: functional interaction networks of proteins, globally integrated and scored. Nucleic Acids Res 39 Database issue D561 D568 21045058 10.1093/nar/gkq973 PMC3013807

[bib25] Chen LH Hsu CY Weng CF 2006 Involvement of P53 and Bax/Bad triggering apoptosis in thioacetamide-induced hepatic epithelial cells. World J Gastroenterol 12 5175 5181 16937528 10.3748/wjg.v12.i32.5175 PMC4088015

[bib26] Huang KW Huang YC Tai KF Chen BH Lee PH Hwang LH 2008 Dual therapeutic effects of interferon-alpha gene therapy in a rat hepatocellular carcinoma model with liver cirrhosis. Mol Ther 16 1681 1687 18665156 10.1038/mt.2008.160

[bib27] Mimeault M Batra SK 2012 Great promise of tissue-resident adult stem/progenitor cells in transplantation and cancer therapies. Adv Exp Med Biol 741 171 186 22457110 10.1007/978-1-4614-2098-9_12 PMC3645316

[bib28] Horie N Pereira MP Niizuma K Sun G Keren-Gill H Encarnacion A 2011 Transplanted stem cell-secreted vascular endothelial growth factor effects poststroke recovery, inflammation, and vascular repair. Stem Cells 29 274 285 21732485 10.1002/stem.584 PMC3524414

[bib29] Ren X Carpenter A Hogaboam C Colletti L 2003 Mitogenic properties of endogenous and pharmacological doses of macrophage inflammatory protein-2 after 70% hepatectomy in the mouse. Am J Pathol 163 563 570 12875976 10.1016/S0002-9440(10)63684-X PMC1868215

[bib30] Kuboki S Shin T Huber N Eismann T Galloway E Schuster R 2008 Hepatocyte signaling through CXC chemokine receptor-2 is detrimental to liver recovery after ischemia/reperfusion in mice. Hepatology 48 1213 1223 18688883 10.1002/hep.22471 PMC2695827

[bib31] Hogaboam CM Bone-Larson CL Steinhauser ML Lukacs NW Colletti LM Simpson KJ 1999 Novel CXCR2-dependent liver regenerative qualities of ELR-containing CXC chemokines. FASEB J 13 1565 1574 10463948 10.1096/fasebj.13.12.1565

[bib32] Luster AD 1998 Chemokines–chemotactic cytokines that mediate inflammation. N Engl J Med 338 436 445 9459648 10.1056/NEJM199802123380706

[bib33] Mantovani A Bonecchi R Locati M 2006 Tuning inflammation and immunity by chemokine sequestration: decoys and more. Nat Rev Immunol 6 907 918 17124512 10.1038/nri1964

[bib34] Gillitzer R Goebeler M 2001 Chemokines in cutaneous wound healing. J Leukoc Biol 69 513 521 11310836

[bib35] Lin Z Rios HF Volk SL Sugai JV Jin Q Giannobile WV 2011 Gene expression dynamics during bone healing and osseointegration. J Periodontol 82 1007 1017 21142982 10.1902/jop.2010.100577 PMC3399909

[bib36] Nedeau AE Bauer RJ Gallagher K Chen H Liu ZJ Velazquez OC 2008 A CXCL5- and bFGF-dependent effect of PDGF-B-activated fibroblasts in promoting trafficking and differentiation of bone marrow-derived mesenchymal stem cells. Exp Cell Res 314 2176 2186 18570917 10.1016/j.yexcr.2008.04.007 PMC2638214

[bib37] Zaja-Milatovic S Richmond A 2008 CXC chemokines and their receptors: a case for a significant biological role in cutaneous wound healing. Histol Histopathol 23 1399 1407 18785122 10.14670/hh-23.1399 PMC3140405

[bib38] Badr G Badr BM Mahmoud MH Mohany M Rabah DM Garraud O 2012 Treatment of diabetic mice with undenatured whey protein accelerates the wound healing process by enhancing the expression of MIP-1a, MIP-2, KC, CX3CL1 and TGF-ß in wounded tissue. BMC Immunol 13 32 22708778 10.1186/1471-2172-13-32 PMC3676145

[bib39] Quandt J Dorovini-Zis K 2004 The beta chemokines CCL4 and CCL5 enhance adhesion of specific CD4+ T cell subsets to human brain endothelial cells. J Neuropathol Exp Neurol 63 350 362 15099025 10.1093/jnen/63.4.350

[bib40] Deshmane SL Kremlev S Amini S Sawaya BE 2009 Monocyte chemoattractant protein-1 (MCP-1): an overview. J Interferon Cytokine Res 29 313 326 19441883 10.1089/jir.2008.0027 PMC2755091

[bib41] Yamashita U Kuroda E 2002 Regulation of macrophage-derived chemokine (MDC, CCL22) production. Crit Rev Immunol 22 105 114 12433129

[bib42] Tymen SD Rojas IG Zhou X Fang ZJ Zhao Y Marucha PT 2013 Restraint stress alters neutrophil and macrophage phenotypes during wound healing. Brain Behav Immun 28 207 217 22884902 10.1016/j.bbi.2012.07.013 PMC3878450

[bib43] Broughton SE Dhagat U Hercus TR Nero TL Grimbaldeston MA Bonder CS 2012 The GM-CSF/IL-3/IL-5 cytokine receptor family: from ligand recognition to initiation of signaling. Immunol Rev 250 277 302 23046136 10.1111/j.1600-065X.2012.01164.x

[bib44] Karimbux NY Saraiya VM Elangovan S Allareddy V Kinnunen T Kornman KS 2012 Interleukin-1 gene polymorphisms and chronic periodontitis in adult whites: a systematic review and meta-analysis. J Periodontol 83 1407 1419 22348697 10.1902/jop.2012.110655

[bib45] Metz CN 2003 Fibrocytes: a unique cell population implicated in wound healing. Cell Mol Life Sci 60 1342 1350 12943223 10.1007/s00018-003-2328-0 PMC11138753

[bib46] Ishida Y Kondo T Kimura A Matsushima K Mukaida N 2006 Absence of IL-1 receptor antagonist impaired wound healing along with aberrant NF-kappaB activation and a reciprocal suppression of TGF-beta signal pathway. J Immunol 176 5598 5606 16622029 10.4049/jimmunol.176.9.5598

[bib47] Rennekampff HO Hansbrough JF Kiessig V Doré C Sticherling M Schröder JM 2000 Bioactive interleukin-8 is expressed in wounds and enhances wound healing. J Surg Res 93 41 54 10945942 10.1006/jsre.2000.5892

[bib48] Schraufstatter IU Trieu K Zhao M Rose DM Terkeltaub RA Burger M 2003 IL-8-mediated cell migration in endothelial cells depends on cathepsin B activity and transactivation of the epidermal growth factor receptor. J Immunol 171 6714 6722 14662875 10.4049/jimmunol.171.12.6714

[bib49] Saraiva M O'Garra A 2010 The regulation of IL-10 production by immune cells. Nat Rev Immunol 10 170 181 20154735 10.1038/nri2711

[bib50] Wynn TA 2003 IL-13 effector functions. Annu Rev Immunol 21 425 456 12615888 10.1146/annurev.immunol.21.120601.141142

[bib51] Muller M Trocme C Lardy B Morel F Halimi S Benhamou PY 2008 Matrix metalloproteinases and diabetic foot ulcers: the ratio of MMP-1 to TIMP-1 is a predictor of wound healing. Diabet Med 25 419 426 18387077 10.1111/j.1464-5491.2008.02414.x PMC2326726

[bib52] Rho SB Chung BM Lee JH 2007 TIMP-1 regulates cell proliferation by interacting with the ninth zinc finger domain of PLZF. J Cell Biochem 101 57 67 17340613 10.1002/jcb.21127

