# Supplementary material for: Exploiting Human CD34+ Stem Cell–conditioned Medium for Tissue Repair
Source: Mol Ther. 2013 Oct 15;22(1):149–59. doi: 10.1038/mt.2013.194 (PMC3978788; doi:10.1038/mt.2013.194)
Supplement: Supplementary Figure S1 — Effect of the expanded CD34+ cells transplanted on damaged rat liver in vivo. [file mt2013194x1.pdf]

Supplementary Figure S1

Effect of the expanded CD34+ cells transplanted on damaged rat liver in vivo. Seven days cultured stem cell population was injected in rats treated with TAA. Liver functions were assessed by albumin (**a**) and total bilirubin (**b**) levels, three days post-treatment. Student's *t* test, *$p = 0.008$; **$p = 0.0063$. The control group (blue colour, n=5) (no TAA), TAA treated group (black colour, n=7), and TAA treated plus cultured stem cells (red colour, n=7). (**c**) Fresh rat liver tissue samples were fixed and stained with hematoxylin/eosin (5 µm sections) for immunohistological analysis. ***ANOVA test (includes all the different groups).

Figure S1

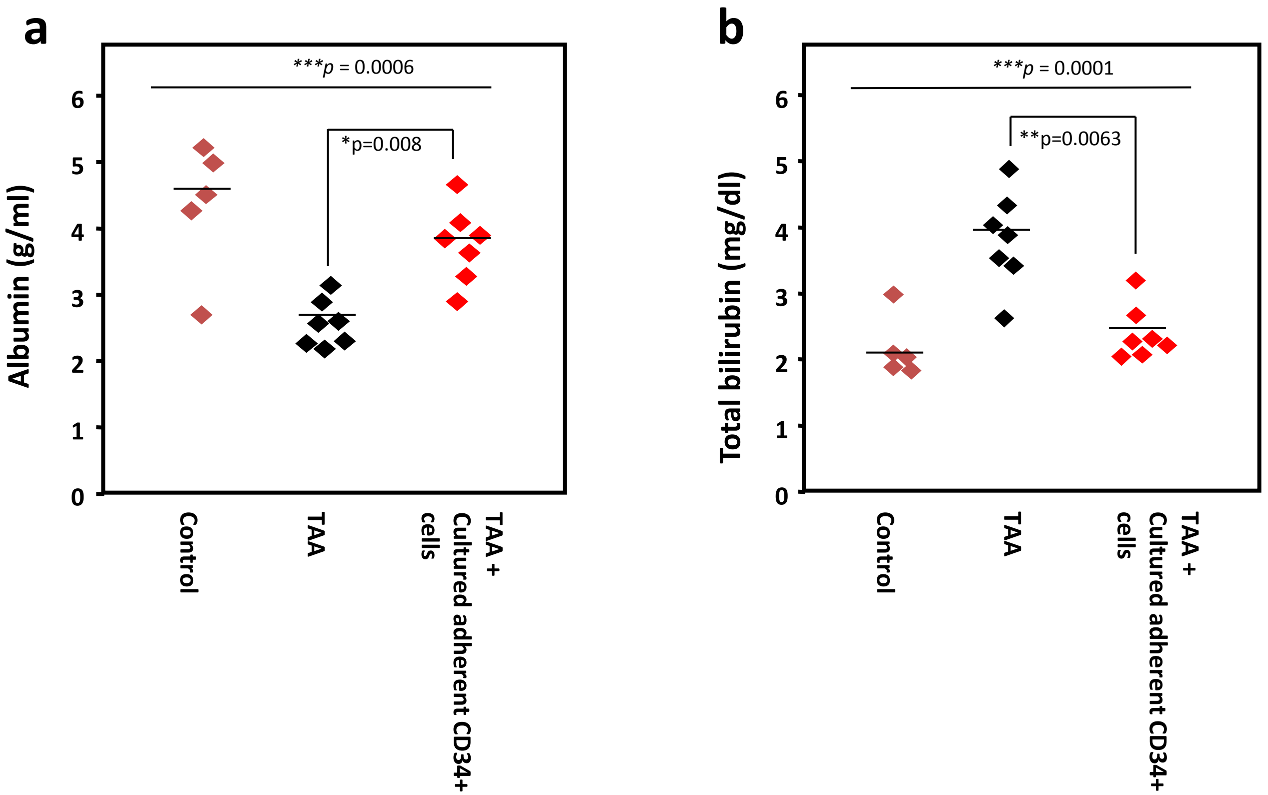

a
***p = 0.0006
*p=0.008
Albumin (g/ml)
0
1
2
3
4
5
6
Control
TAA
TAA + Cultured adherent CD34+ cells
b
***p = 0.0001
**p=0.0063
Total bilirubin (mg/dl)
0
1
2
3
4
5
6
Control
TAA
TAA + Cultured adherent CD34+ cells

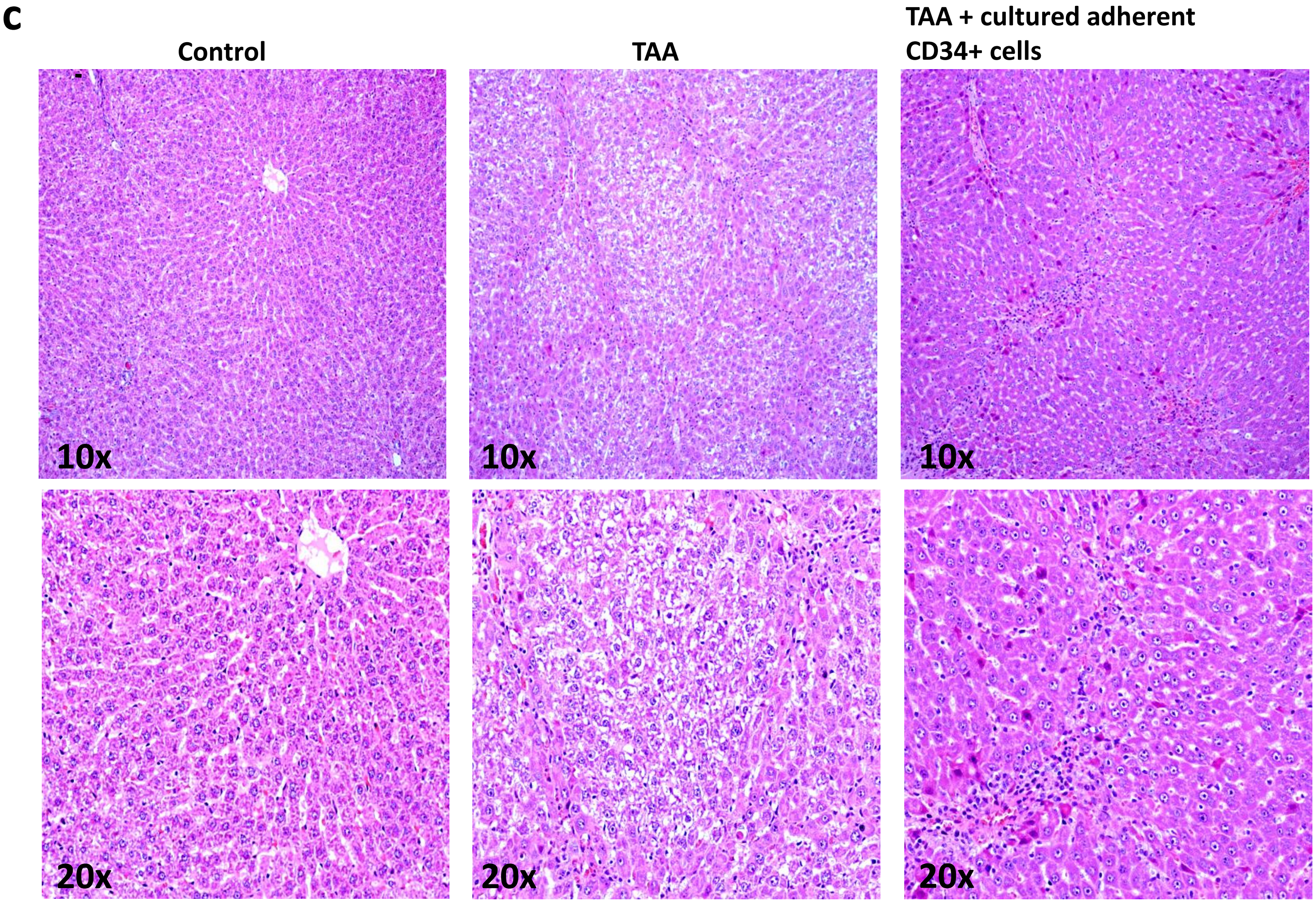
c
Control
TAA
TAA + cultured adherent CD34+ cells
10x
10x
10x
20x
20x
20x
